# Prevalence of Vitamin D Hypovitaminosis in Croatia: A Cross-Sectional Study Based on Routine Clinical Examinations

**DOI:** 10.3390/nu17243863

**Published:** 2025-12-11

**Authors:** Jelena Kelemen, Luka Bulić, Petar Brlek, Renata Zadro, Jelena Pavlović, Marin Vučić, Eva Brenner, Dragan Primorac

**Affiliations:** 1 St. Catherine Specialty Hospital, 10000 Zagreb, Croatialuka.bulic@svkatarina.hr (L.B.);; 2 School of Medicine, Josip Juraj Strossmayer University of Osijek, 31000 Osijek, Croatia; 3 Department of Molecular Biology, Faculty of Science, University of Zagreb, 10000 Zagreb, Croatia; 4 Faculty of Pharmacy and Biochemistry, University of Zagreb, 10000 Zagreb, Croatia; 5 Faculty of Dental Medicine and Health, Josip Juraj Strossmayer University of Osijek, 31000 Osijek, Croatia; 6 Forensic Science Program, Department of Biochemistry & Molecular Biology, The Pennsylvania State University, State College, PA 16802, USA; 7 The Henry C. Lee College of Criminal Justice and Forensic Sciences, University of New Haven, West Haven, CT 06516, USA; 8 Sana Kliniken Oberfranken, 96450 Coburg, Germany; 9 School of Medicine, University of Split, 21000 Split, Croatia; 10 Medical School, University of Rijeka, 51000 Rijeka, Croatia; 11 Medical School, University of Mostar, 88000 Mostar, Bosnia and Herzegovina; 12 National Forensic Sciences University, Gandhinagar 382007, India; 13 School of Medicine, University of Pittsburgh, Pittsburgh, PA 15213, USA

**Keywords:** hypovitaminosis, vitamin D, population study, immune system function, musculoskeletal health, public health

## Abstract

**Background/Objectives**: Vitamin D deficiency is recognized as a global public health concern due to its implications for bone health, immune regulation, and chronic disease risk. Despite its significance, comprehensive data on the prevalence of hypovitaminosis D in the Croatian population remain limited. This study aimed to determine the distribution of 25-hydroxyvitamin D (25-OH D) levels in a group of patients who underwent routine clinical examination, evaluate the prevalence of deficiency, and assess potential associations with demographic factors such as age and sex. **Methods**: This cross-sectional study included 829 individuals aged 19–85 years who underwent routine clinical testing at our institution. Serum 25-OH D concentrations were measured and classified as normal (≥75 nmol/L), deficient (<75 nmol/L, ≥50 nmol/L), or severely deficient (<50 nmol/L). Data on age and sex were extracted, and statistical analyses included descriptive statistics, tests for normality (Kolmogorov–Smirnov), comparisons (Mann–Whitney U, Kruskal–Wallis), and correlation testing (Spearman’s rho). Significance was set at *p* < 0.05. **Results**: The cohort consisted of 525 females (63.3%) and 304 males (36.7%), with a mean age of 49.2 ± 15.8 years. The mean and median serum 25-OH D concentrations were 53.5 and 53.0 nmol/L, respectively (IQR: 40.0–65.0). Severe deficiency (<50 nmol/L) was present in 43.7% of participants, while an additional 49.2% exhibited moderate deficiency, leaving only 7.1% with sufficient levels. No statistically significant differences in vitamin D levels were observed between sexes, nor was there a significant correlation between age and vitamin D concentration (*p* > 0.05). **Conclusions**: Vitamin D deficiency is highly prevalent in the Croatian population, with more than 90% of individuals showing suboptimal serum levels. The absence of significant associations with age or sex suggests a widespread deficiency pattern, underscoring the need for nationwide preventive strategies, including dietary supplementation and public health education initiatives to improve vitamin D status.

## 1. Introduction

**Figure 1:**
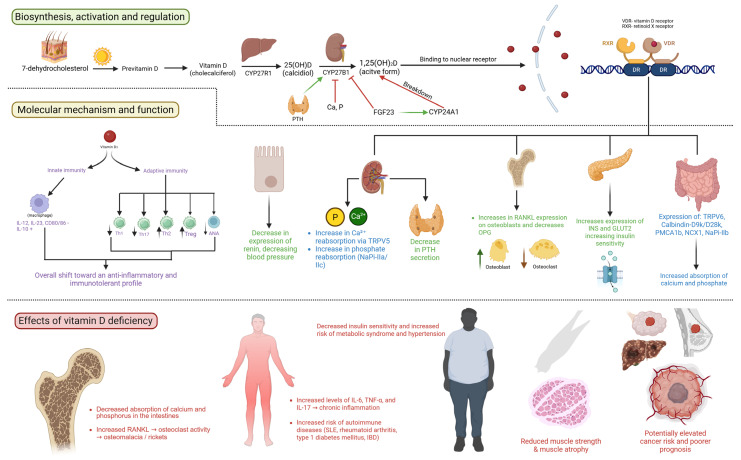
Biosynthesis and effector pathways of vitamin D with consequences of hypovitaminosis (created with Biorender.com). The first section of the image shows the pathway of vitamin D synthesis, including previtamin D, cholecalciferol, and active form, as well as binding to the nuclear receptor. The second section of the image shows its physiological functions, including skeletal, gastrointestinal, renal, thyroid, and immune system effects. Finally, the third section shows the effects of deficiency, including musculoskeletal and inflammatory health issues. VDR—vitamin D receptor, RXR—retinoid X receptor, PTH—parathyroid hormone, Th—T-helper cell, SLE—systemic lupus erythematosus, IBD—inflammatory bowel disease, IL—interleukin, TNF—tumor necrosis factor, RANKL—receptor activator of nuclear factor kappa-B ligand.

Vitamin D, also known as the “sunshine vitamin”, is an important factor in human health. Its nickname stems from the essential role sunlight has in its biosynthesis, which occurs when ultraviolet B radiation turns 7-dehydrocholesterol into cholecalciferol or vitamin D3. Subsequently, cholecalciferol is turned into 25-hydroxyvitamin D via the enzyme vitamin D-25-hydroxylase, a process that takes place in the liver. The reaction is stimulated by parathyroid hormone (PTH). The final step occurs in the kidney, where 25-OH D becomes 1,25-(OH)_2_ D—the most active form of vitamin D [1]. However, apart from the aforementioned biosynthesis pathway, an alternative way to take in vitamin D is by using dietary supplements which contain it in high concentrations. Vitamin D is a liposoluble steroid compound, most widely known for its role in phosphorus and calcium homeostasis [2]. The mechanisms by which this is achieved are the increase in intestinal absorption and the reduction in renal secretion, which is extremely important for bone homeostasis and regulating bone resorption or deposition depending on the concentration of those ions. However, it also regulates a wide range of other processes, some of which are crucial for the normal function of the immune system. Vitamin D has both genomic and non-genomic functions. When it binds to the vitamin D receptor (VDR), that complex migrates from the cytoplasm to the nucleus, where, along with the retinoid X receptor (RXR), it binds to its designated place on the DNA strand [3]. The non-genomic functions are mediated through the activation of signaling molecules like phospholipases, second messengers, certain protein kinases, and even the opening of various ion channels [4] (Figure 1).

Recent studies have found that vitamin D has many health benefits which supersede regulating serum levels of calcium and phosphorus. For example, it has been established that vitamin D aids in combating cancer-related fatigue, especially in those who had a previous vitamin D deficiency (VDD) [5]. Additionally, one of the most recently discussed functions of vitamin D is its immunomodulatory capabilities. Vitamin D plays a crucial role in both innate and adaptive immunity. It has been shown to regulate inflammation and immune balance, while its deficiency can play a role in autoimmune disease, chronic inflammation, and even some forms of cancer. Furthermore, one study demonstrated that vitamin D can potentially impact the treatment of hepatocellular carcinoma by suppressing angiogenesis, inducing apoptosis or reducing the amount of pro-inflammatory cytokines being released [6]. Another important and often overlooked use for vitamin D is its effects on insulin sensitivity and blood glucose levels. A study has found that vitamin D plays a role in both type 1 and type 2 diabetes mellitus, established through regulation of inflammation and the enhancement of GLUT4 concentration, while also simultaneously increasing insulin gene expression. Though other studies and effects of vitamin D over time on blood glucose levels are needed to confirm these findings [7].

Despite all of its benefits, vitamin D deficiency has continued to be a highly prevalent public health concern in most modern societies. A serum concentration of 75 to 125 nmol/L is considered to be sufficient for most people, while anything less than 50 nmol/L can greatly increase the risk of disease according to the National Institutes of Health (NIH). When reviewing the dates of death in the United States between 1979 and 2004, death rates were higher in the winter than summer. Interestingly, it is said that a significant proportion of those deaths could have potentially been prevented had the deceased kept higher 25-OH vitamin D levels [8]. This information once again demonstrates that keeping serum levels of vitamin D in its normal range is crucial for overall health. The effects of VDD on bone health have long been known. For example, VDD can worsen the symptoms of osteoarthritis by increasing the number of matrix metalloproteinases, which are active. Vitamin D deficiency is also associated with osteoporosis. VDD disturbs calcium homeostasis, which leads to secondary hyperparathyroidism, causing osteoporosis and bone loss [9].

Although vitamin D plays diverse physiological roles, the central epidemiological concern in public health is the widespread prevalence of hypovitaminosis D across Europe. Numerous population-based studies have shown that countries at mid-to-high latitudes experience substantial seasonal variability in serum 25-hydroxyvitamin D (25(OH)D), with deficiency rates higher during winter months [10,11,12,13]. Despite high annual sunshine hours in coastal regions (such as Croatia), recent analyses suggest that lifestyle patterns, particularly increased indoor work, reduced sun exposure, and low consumption of vitamin D–rich foods, may contribute to lower-than-expected population vitamin D levels [14]. Existing data on vitamin D status in Croatia are sparse, fragmented, and often limited to specific subgroups such as children, older adults, or patients with chronic disease. No recent, large-scale dataset has described the distribution of 25(OH)D levels among adults undergoing routine testing, nor have prior studies systematically assessed deficiency categories using standardized thresholds. Additionally, unlike several northern European countries, Croatia does not have mandatory fortification of commonly consumed foods [15].

The aim of this study was to investigate and assess the prevalence of vitamin D hypovitaminosis in the Croatian population. To our knowledge, this is the largest dataset of routine clinical 25(OH)D measurements in our population, and its results may provide valuable insights into the public health situation regarding this crucial nutrient.

## 2. Materials and Methods

### 2.1. Study Design and Participants

The conducted study had a cross-sectional design, involving a convenience sample of individuals who attended a routine clinical checkup at our institution. The participants were sampled throughout all seasons of the year, from 2021 to 2025. The parameter of interest was 25-OH vitamin D, measured from the participants’ blood samples as part of the examination. The data were retrospectively extracted from our clinical database and included patient age (expressed in years), sex (male or female), and 25-OH vitamin D serum levels (expressed in nmol/L). Exclusion criteria were ages under 18 or over 85, and the diagnosis of a chronic illness which significantly impacts serum vitamin D levels (such as chronic liver or kidney disease), previously made by a medical specialist and stated in the patient history documentation. Based on the level of vitamin D deficiency, the population was divided into three categories: Normal value (≥75 nmol/L), Deficiency (<75 nmol/L, ≥50 nmol/L), and Severe deficiency (<50 nmol/L), with cutoff values in line with those in previously published literature [8,16]. The study was approved by our institutional ethics committee.

### 2.2. Biochemical Analysis Specifications

For the quantitative determination of 25-hydroxyvitamin D in serum samples, the ARCHITECT 25-OH Vitamin D 5P02 assay (Abbott Laboratories, Chicago, IL, USA) was used. The method is based on chemiluminescent microparticle immunoassay (CMIA) technology. All reagents, calibrators, and controls were prepared and handled according to the manufacturer’s recommendations. The reagent kit included anti-vitamin D IgG–coated paramagnetic microparticles in MES buffer with ProClin 300 preservative, an acridinium-labeled vitamin D conjugate in MES buffer with surfactant and sodium azide, and assay diluent consisting of citrate buffer with EDTA, methanol, 8-anilino-1-naphthalenesulfonic acid (ANSA), surfactant, and ProClin 300 K153375. Calibration of the analytical system was performed using ARCHITECT 25-OH Vitamin D 5P02 Calibrators (Abbott Laboratories, Chicago, IL, USA). The calibration set consisted of six levels (A–F), each containing PBS buffer and human serum, with 25-OH vitamin D concentrations ranging from 0.0 nmol/L to 400.0 nmol/L. The calibrators contained ProClin 950 and sodium azide as preservatives, and assay standardization was traceable to NIST SRM 2972 reference material K153375. Analytical performance was monitored using Technopath Clinical Diagnostic Multichem IA Plus 05P76-10 (Technopath Manufacturing Ltd., Ballina, Co. Tipperary, V94 N248, Ireland) at three concentrations: low, medium and high. The controls were prepared in PBS buffer with human serum and preserved with ProClin 950 and sodium azide. Target ranges were applied as specified by the manufacturer. All samples were analyzed on the ARCHITECT ci4100 immunochemistry system following the manufacturer’s validated operating protocol. The instrument automatically calculated results based on the established six-point calibration curve. Quality control procedures at our institution are regulated by the Joint Commission International (JCI) Accreditation for hospitals.

### 2.3. Statistical Analysis

Descriptive statistical analysis of 25-OH vitamin D levels included the determination of the population mean and standard deviation, as well as the median, interquartile range (IQR), 95%-confidence interval, and minimum-maximum range. Additionally, normality testing was done for the vitamin D and age parameters using the Kolmogorov–Smirnov test. Population distributions were graphically shown using histograms. Comparative statistical analysis involved the comparison of categorical and continuous distributions between groups, as well as correlation testing. When comparing distributions between two unrelated groups, the t-test and Mann–Whitney U-test were used for normally and non-normally distributed data, respectively. When comparing distributions between three or more unrelated groups, the ANOVA and Kruskal–Wallis tests were used for normally and non-normally distributed data, respectively. Comparison across categorical variables was done using the Chi-squared test. Finally, the significance of a correlation between two continuous variables was determined using Pearson’s r and Spearman’s rho for normally and non-normally distributed data, respectively. For both descriptive and comparative statistical tests, a result was considered statistically significant for a *p*-value less than 0.05.

## 3. Results

### 3.1. Descriptive Statistics

**Figure 2:**
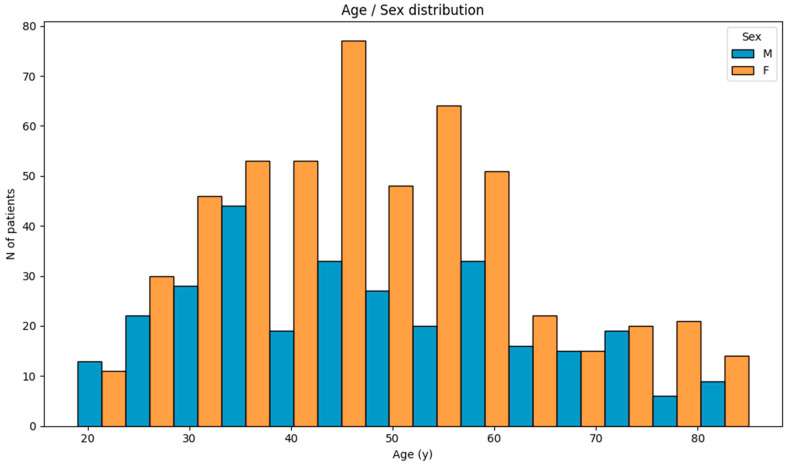
Distribution of study participants based on age and sex.

This cross-sectional analysis involved 829 individuals from the Croatian population. Initially, the distribution of the cohort was analyzed based on age and sex, including normality testing (Figure 2). Based on age, the participants involved in the study ranged from 19 to 85 years of age. Normality testing showed a statistically significant deviation from the normal distribution (Kolmogorov–Smirnov test, *p* = 0.001). Regarding sex, out of the total 829 participants, 525 were female and 304 were male.

**Figure 3:**
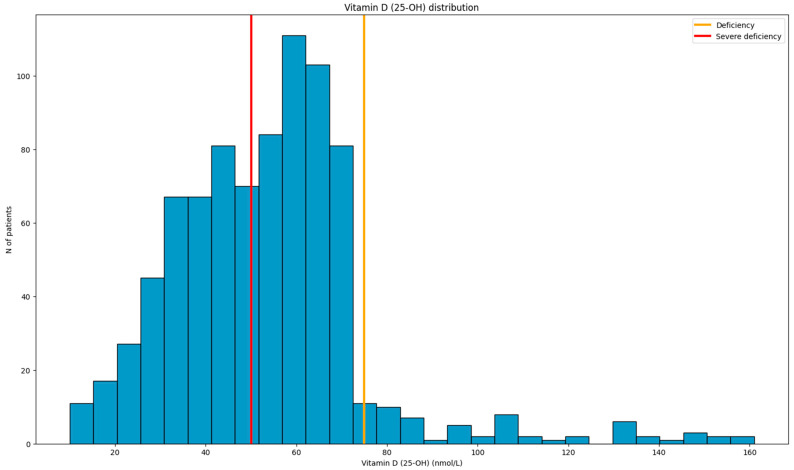
Distribution of vitamin D (25-OH) levels.

After population characterization based on age and sex, the distribution of vitamin D (25-OH) was determined and compared with reference values for deficiency and severe deficiency (Figure 3). The population mean and median were 53.5 and 53.0, respectively, with a standard deviation of 21.6 and an interquartile range from 40.0 to 65.0. The complete range of values was from 10.0 to 161.0, with a 95%-confidence interval from 52.1 to 55.0. Normality testing revealed a statistically significant deviation from the normal distribution (Kolmogorov–Smirnov test, *p* < 0.001).

Among the categories describing the severity of vitamin D deficiency, including normal value, deficiency, and severe deficiency, the number of participants was 59, 408, and 362, respectively.

### 3.2. Comparative Statistics

**Figure 4:**
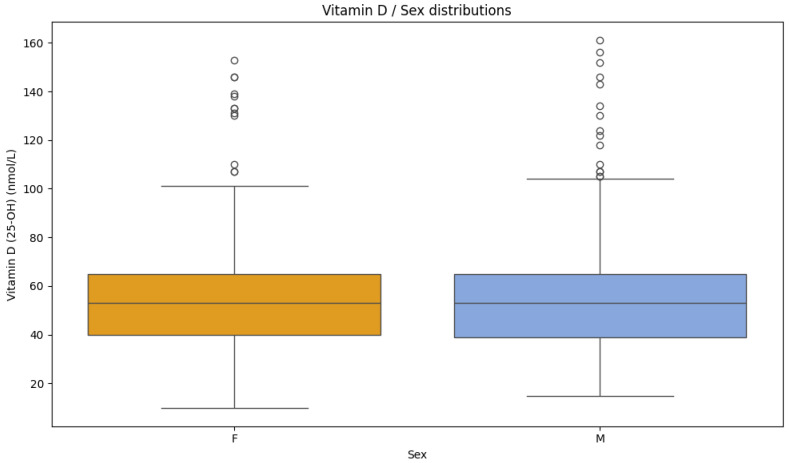
Vitamin D level distributions for the female (F) and male (M) sexes (Mann-Whitney U-test, *p* = 0.560).

25-OH vitamin D levels were compared between male and female participants in order to determine distribution differences. When compared statistically, there was no significant difference between male and female participants (Figure 4).

**Figure 5:**
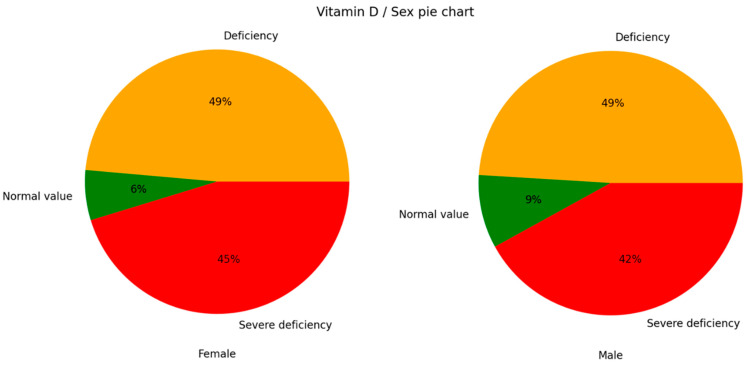
Vitamin D categories for the male and female sexes (Chi-square test, *p* = 0.305).

Additionally, categorical vitamin D distributions were also compared between the male and female sexes. The male population contained a slightly greater percentage of individuals with normal values, while the female population contained a slightly greater percentage of individuals with severe deficiency. However, there was no statistically significant difference between the two subgroups (Figure 5).

**Figure 6:**
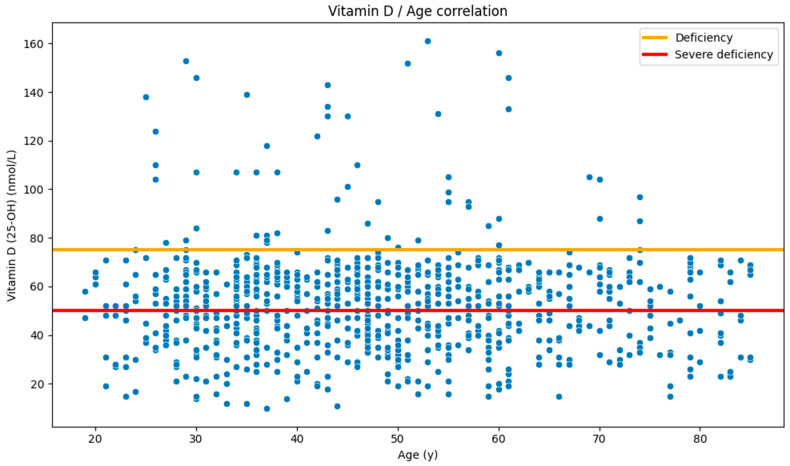
Age and vitamin D (25-OH) level scatter plot (Spearman’s rho: *p* = 0.466).

Apart from sex, distribution comparisons and correlational analyses were also done with regard to participant age. Correlational analysis showed no statistically significant correlation between age and 25-OH vitamin D levels (Figure 6).

**Figure 7:**
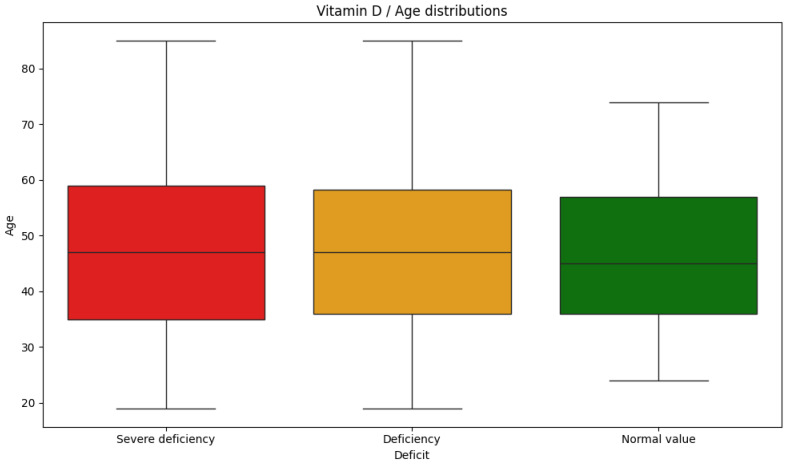
Age distributions for vitamin D categories (Kruskal-Wallis H-test, *p* = 0.604).

Additionally, differences in age distribution were assessed between the three vitamin D categories, and no statistically significant differences in age distribution were found (Figure 7).

## 4. Discussion

### 4.1. Comparison with Other Population Studies

The presented results of our cross-sectional analysis represent one of the most extensive assessments of vitamin D status in Croatia, conducted on a group of individuals reporting for routine clinical examination. The results reveal a pronounced prevalence of vitamin D deficiency, with nearly 44% of participants showing severe deficiency (deficiency) (<50 nmol/L) and an additional 49% classified as deficient (insufficient) (<75 nmol/L). These findings are consistent with reports from other large-scale vitamin D population studies.

The study published by Diaz-Rizzolo et al. investigated the prevalence of vitamin D deficiency in Catalonia, another region in the Mediterranean area [16]. The study was conducted on over half a million participants, including patients over 18 years of age. The thresholds set by the authors were 20 and 30 ng/mL for deficiency and insufficiency, respectively, which roughly corresponds to our thresholds. Results showed that around 80% of the younger population had suboptimal levels, which the authors described as paradoxical due to sunlight exposure in the Mediterranean climate. Gromova et al. conducted a population study in Kazakhstan, which involved over 1300 participants [17]. The results demonstrated a median 25-OH D level below 20 ng/mL (or 50 nmol/L). A review article by Man et al. included ten cross-sectional studies conducted on the Chinese population [18]. Out of the ten, five showed a mean 25-OH D level below 50 nmol/L. A clinically specific population was evaluated by Hoxha et al., including patients undergoing total shoulder or elbow arthroplasty [19]. The study separately analysed patients who received vitamin D supplementation and those who did not. Remarkably, 87% of patients who did not report a regular vitamin D intake showed low serum vitamin D levels. Additionally, in the group that reported supplementation, 38% still showed low serum vitamin D levels. Similar to the aforementioned findings, vitamin D hypovitaminosis has been observed by many more studies across different populations, demonstrating the significance of this public health issue on a global scale [20,21,22,23,24].

Several factors may contribute to the high prevalence of deficiency observed in Croatia. Firstly, while the geographic position of the country provides substantial sunlight exposure, behavioral and cultural factors such as indoor lifestyles and extensive sunscreen use can affect cutaneous synthesis. A study by Passeron et al. demonstrated that high sun-protection factor sunscreens, combined with limited exposure to sunlight, can compromise serum vitamin D levels [25]. Secondly, serum levels of vitamin D largely depend on dietary culture, as well as varying country-specific vitamin D food fortification policies. This is well-highlighted in the review article by Niedermaier et al., which states that vitamin D fortification prevents an estimated 11,000 cancer deaths in the European Union annually, as well as 27,000 annual cancer deaths across all European countries. Furthermore, it is estimated that the number of prevented annual cancer deaths in the European Union would increase tenfold if the countries in question were to implement optimal fortification policies [15].

In the context of global vitamin D research, our results fall in line with others that have established hypovitaminosis D in their respective populations. The lack of significant sex- or age-related differences further suggests that the deficiency affects both younger and older adults uniformly, emphasizing the systemic nature of the issue. While some articles have reported different deficiency rates based on age and sex, our results do not fall in line with these trends [20,26].

### 4.2. Clinical Implications and Public Health Relevance

The high prevalence of vitamin D deficiency observed in this cohort may have important clinical consequences. Vitamin D plays a central role in calcium and phosphate homeostasis, and chronic deficiency reduces calcium absorption, triggers secondary hyperparathyroidism, and accelerates bone turnover, increasing the risk of osteopenia, osteoporosis, and fragility fractures [27,28]. Given that over 90% of individuals in this sample had suboptimal values, these musculoskeletal risks may not be limited to older adults but could also affect younger age groups, potentially influencing long-term bone health. Vitamin D deficiency has also been linked to impaired skeletal muscle performance through VDR-mediated pathways and mitochondrial dysfunction, contributing to muscle weakness and reduced physical function [29]. Evidence suggests that supplementation in deficient individuals can improve muscle performance and mobility, indicating that low vitamin D levels could contribute to functional limitations even in otherwise healthy adults [30]. Beyond musculoskeletal effects, vitamin D has recognized immunomodulatory properties. Active 1,25-(OH)_2_D regulates cytokine production and promotes immune tolerance via VDRs expressed in immune cells [31]. For this reason, the association between vitamin D deficiency and respiratory infections, particularly COVID-19, has been postulated in the literature [32,33]. For example, it has been shown that severe COVID-19 patients had lower serum vitamin D levels when compared to mild cases [34]. The high prevalence of deficiency in this sample may reflect an increased vulnerability to seasonal infectious diseases. Associations between vitamin D deficiency and autoimmune conditions such as multiple sclerosis, rheumatoid arthritis, inflammatory bowel disease, and systemic lupus erythematosus have also been reported, potentially due to effects on regulatory T cell function [35,36]. Although causation cannot be inferred, the extent of deficiency observed suggests that this may be a relevant contributing factor in immune-mediated disease burden. Vitamin D has additionally been implicated in cardiovascular and metabolic regulation. Low serum 25-OH vitamin D concentrations have been associated with hypertension, endothelial dysfunction, increased cardiovascular mortality, and metabolic disturbances such as insulin resistance, increased adiposity, and a higher risk of metabolic syndrome and type 2 diabetes mellitus [37,38]. While the present study did not assess these outcomes, the degree of deficiency underscores the need for future research evaluating these associations within the Croatian population.

An increasingly talked-about role of vitamin D in cancer is a noteworthy point in this discussion. The scientific literature highlights the role of vitamin D in the progression of malignant diseases, directly through the regulation of tumor cell proliferation and apoptosis, as well as indirectly through immune system regulation [39]. The association between vitamin D serum levels and risk of cancer development remains a debate, with different studies reporting associations of varying significance for specific cancer types [40,41]. Similarly, correlations between vitamin D serum levels and malignant disease prognosis have also proven inconsistent, requiring further investigation [42]. Still, the supportive role of vitamin D in cancer patient management cannot be overlooked. Considering the higher cancer death rate in Croatia compared to the European Union, this point warrants further investigation in the Croatian population [43]. Additionally, it is also necessary to highlight the underlying role of genetics in vitamin D deficiency [44,45]. Understanding these genetic determinants may help stratify individuals at higher risk for deficiency and guide personalized supplementation strategies. In line with this reasoning, a genomic population-based analysis of vitamin D-relevant loci in Croatia is warranted and might provide further valuable insights into the background of the results presented in this article.

### 4.3. Study Limitations

This study has several aspects that should be considered when interpreting its findings. Firstly, the study included a large and clinically relevant number of participants, which represents a notable strength. The sample consisted of individuals undergoing standard routine health examinations at a single healthcare institution, and although patients came from various regions of Croatia and were not selectively recruited, the extent to which the sample reflects the broader national population remains uncertain. Secondly, information on the season or month of blood collection was not available, which limits the ability to relate measured 25(OH)D concentrations to potential seasonal patterns. Thirdly, data on additional factors that may influence vitamin D status (e.g., BMI, dietary intake, supplementation, or sun-exposure habits) were not collected. While these parameters were outside the primary aims of the study, their absence may reduce comparability with cohorts where such information is reported. Finally, as the analysis was conducted within a single healthcare setting, the findings may not fully reflect the diversity of patient demographics or healthcare utilization patterns across the entire population.

## 5. Conclusions

This cross-sectional analysis revealed a strikingly high prevalence of vitamin D deficiency among individuals who underwent a routine clinical examination, with over 90% of individuals exhibiting suboptimal serum 25-OH vitamin D levels. The absence of significant differences across sex and age groups suggests that hypovitaminosis D represents a population-wide issue rather than one confined to specific demographic subgroups. These findings underscore the urgent need for public health measures aimed at improving vitamin D status, including education on sunlight exposure, fortification strategies, and supplementation programs. Future studies should employ probabilistic sampling frameworks, incorporate seasonal stratification and detailed lifestyle data, and perform multivariate modeling to better understand determinants of vitamin D status and accurately estimate prevalence at the population level.

## Data Availability

The original contributions presented in this study are included in the article. Further inquiries can be directed to the corresponding author.
